# Electro-optical interfacial effects on a graphene/π-conjugated organic semiconductor hybrid system

**DOI:** 10.3762/bjnano.9.90

**Published:** 2018-03-23

**Authors:** Karolline A S Araujo, Luiz A Cury, Matheus J S Matos, Thales F D Fernandes, Luiz G Cançado, Bernardo R A Neves

**Affiliations:** 1 Departamento de Física - Universidade Federal de Minas Gerais – UFMG, Belo Horizonte, Brazil; 2 Instituto Federal de Educação, Ciência e Tecnologia de Minas Gerais – IFMG, Ponte Nova, Brazil; 3 Universidade Federal de Ouro Preto – UFOP, Ouro Preto, Brazil

**Keywords:** DFT calculations, graphene, organic semiconductors, scanning probe microscopy, self-assembly

## Abstract

The influence of graphene and retinoic acid (RA) – a π-conjugated organic semiconductor – interface on their hybrid system is investigated. The physical properties of the interface are assessed via scanning probe microscopy, optical spectroscopy (photoluminescence and Raman) and ab initio calculations. The graphene/RA interaction induces the formation of a well-organized π-conjugated self-assembled monolayer (SAM) at the interface. Such structural organization leads to the high optical emission efficiency of the RA SAM, even at room temperature. Additionally, photo-assisted electrical force microscopy, photo-assisted scanning Kelvin probe microscopy and Raman spectroscopy indicate a RA-induced graphene doping and photo-charge generation. Finally, the optical excitation of the RA monolayer generates surface potential changes on the hybrid system. In summary, interface-induced organized structures atop 2D materials may have an important impact on both design and operation of π-conjugated nanomaterial-based hybrid systems.

## Introduction

Organic semiconductors offer a wide range of possible applications, from thin-film transistors to sensors and solar cells [1–6]. Their optical and electronic properties are strongly linked to intermolecular interaction parameters associated with molecular packing and/or ordering [7]. Previous works have demonstrated that intermolecular interactions can dramatically reduce the luminescence quantum yield in solid-state devices [8–13]. In this context, it is important to control the ordering of π-conjugated organic molecules to make their use on optoelectronic devices possible. In another front, self-assembled monolayers (SAMs) on low-dimensional systems, such as a graphene (an archetypical 2D material), have attracted significant attention due to the myriad of applications in nanoelectronic devices [14–16]. Aiming at the improvement of graphene-based devices, SAM surface functionalization is employed for both doping level control and work function tuning [14–16]. The present work brings these two fronts together by investigating a graphene/retinoic acid (RA) – a π-conjugated organic semiconductor – hybrid system and its interfacial effects. Scanning probe microscopy (SPM) and Raman scattering experiments, along with first-principles calculations, reveal the presence of a highly ordered RA self-assembled monolayer atop graphene and graphite. The electro-optical characterization of the hybrid system discloses interfacial influences: graphene-promoted high photoluminescence efficiency of RA and RA-induced doping and charge modulation of graphene. The results suggest that low-dimensional hybrid systems, with reciprocal modifications of the constituents’ original properties, may tailor the desired properties in future device applications.

## Results and Discussion

**Figure 1:**
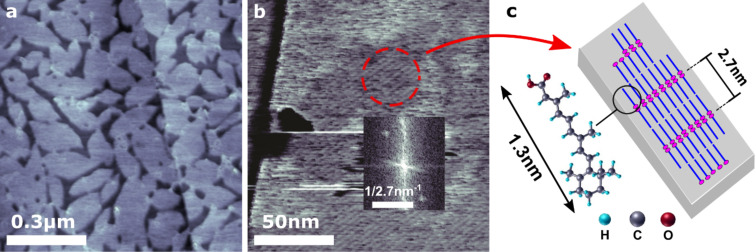
Morphological and structural characterization of the retinoic acid (RA)–graphene hybrid system. (a) Atomic force microscopy topographic image of a RA self-assembled monolayer, partially covering a graphite microplate substrate. (b) High-resolution AFM image (adhesion channel in peak force mode – see Experimental section) of the RA monolayer. The inset shows its fast Fourier transform, evidencing well-defined periodical RA ripples (periodicity: 2.7 ± 0.1 nm). (c) Schematic representation of RA molecule, which is 1.3 nm long, and its proposed configuration atop graphene. The dashed red circle in b) highlights the self-assembled structure and enables a direct comparison with (c).

The first goal of this work was to investigate whether interfacial interactions could lead to the formation of an organized RA 2D monolayer-type film on a supporting substrate. Standard smooth substrates like mica, silicon oxide (SiO*_x_*) on Si, graphene and graphite microplates were tested (see Experimental section for a definition of graphite microplates). Several attempts with mica and SiO*_x_* yielded amorphous 3D-like RA agglomerates only (Figure S1, Supporting Information File 1). However, the graphene-RA interface in graphite microplate substrates systematically produced ultrathin 2D-like RA films. Figure 1 summarizes the morphological characterization of retinoic acid self-assembled films on graphite microplates prepared via spin-coating (see Experimental section). Since the adsorbing species interaction with monolayer graphene can be largely influenced by the underlying substrate [17–18], we expect that graphite microplates would screen any spurious influence of a given supporting substrate on the adsorbing RA molecule. In other words, we expect that a graphite flake (formed by tens or hundreds of graphene layers), rather than monolayer graphene on a Si/SiO*_x_* substrate, for example, should enable a true RA/graphene interfacial interaction, free from deleterious influence of the supporting substrate. A free-standing monolayer graphene membrane would also avoid spurious substrate influences, but this kind of sample would bring some obstacles for SPM experiments and it was not available in the present work.

Therefore, Figure 1a shows a graphite microplate partially covered by an ultrathin (0.4 nm-thick) RA self-assembled monolayer. (See Figure S2a in Supporting Information File 1 for a typical topographic image of a pristine graphite microplate). The spatial ordering of such RA SAM is evidenced in Figure 1b, which shows periodically spaced ripples within the monolayer that are verified by the fast Fourier transform image inset. Both images in Figure 1b clearly indicate a 2.7 nm structural periodicity. This value is about twice the length of a RA molecule, and enables a possible structural model for the RA SAM, with RA molecules horizontally aligned on the graphene surface, as illustrated in the schematic model of Figure 1c.

**Figure 2:**
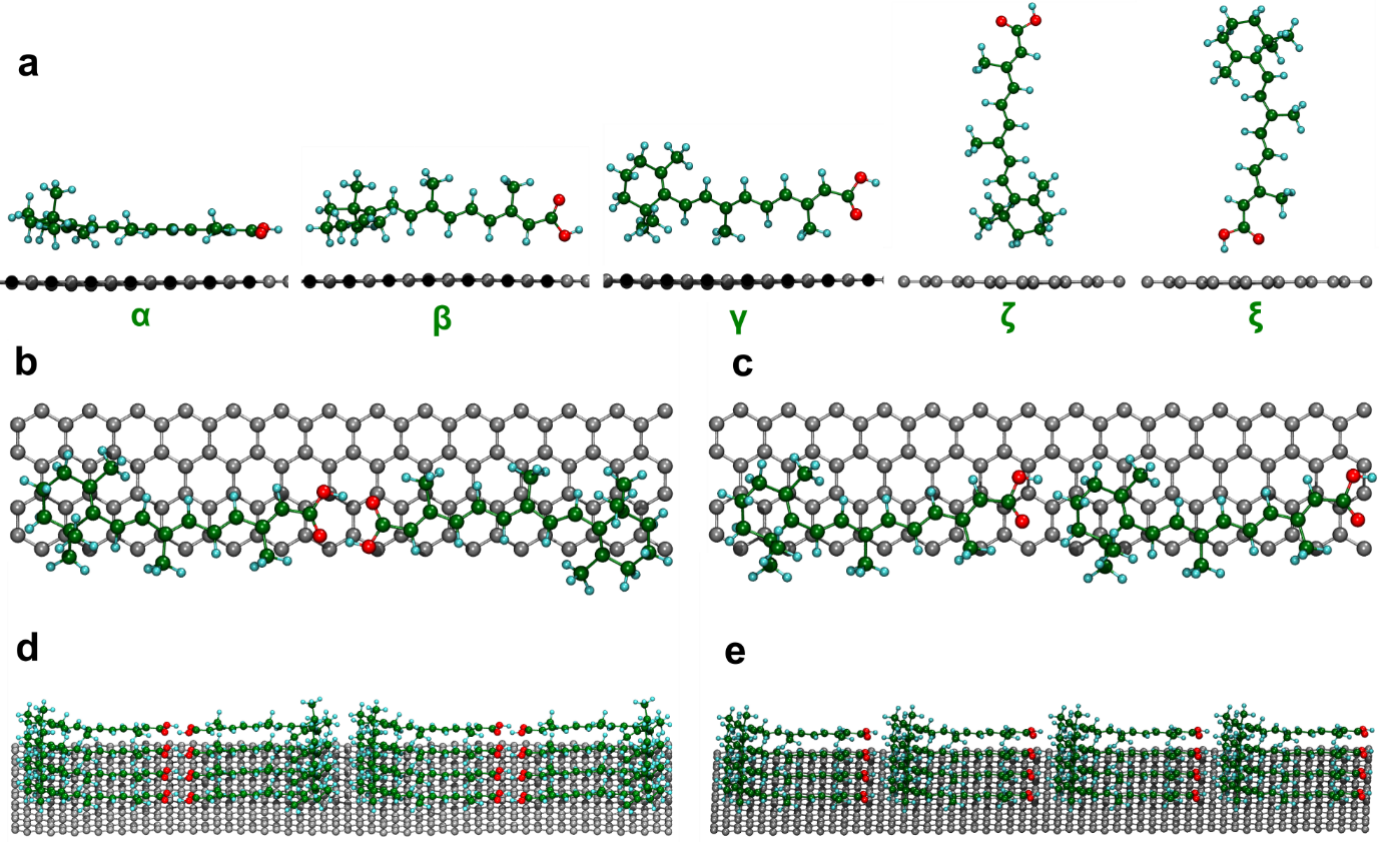
Calculated structures of the RA/graphene hybrid system. (a) Five different unit cell configurations of the RA/graphene system. (b)-(c) Top and (d)-(e) lateral views of the most stable molecular configuration α with two possible pair structures along the chain axis: tail–tail (b) and (d) and tail–head (c) and (e) configurations.

In order to confirm the nature of RA rippled domains seen in Figure 1, possible structures of RA–graphene were analyzed by first-principles calculations (Figure 2). Five different molecular configurations for a single RA molecule atop graphene were considered, labeled as α, β, γ, ζ and ξ (see Figure 2a). The energetics studies for these structures pointed to α as the most stable configuration. In other words, the RA molecule rests horizontally on the graphene surface, with its aromatic ring and backbone planes parallel to the graphene surface. Based on this most stable configuration for isolated RA molecules, the interaction between RA pairs on graphene was also investigated, as shown in Figure 2b–e. In the first configuration (Figure 2b – top view), both OH and O molecular terminations at the RA tail interact with its neighboring molecule counterparts forming hydrogen bonds. This is called a tail–tail configuration. In an additional step, the RA molecules are allowed to interact laterally with other pairs and their carbon ring heads face each other, forming rows of RA dimers (Figure 2d – side view). In the other configuration (tail–head), the tail of one molecule interacts with the carbon ring head of another (Figure 2c – top view and Figure 2e – side view). Comparing the total energy involved in both tail–tail and tail–head configurations, the former was found to be the most stable by 0.5 eV, indicating that hydrogen bond formation minimizes the system energy [16]. This result corroborates the experimental results in Figure 1 for the structure of the graphene-RA hybrid system, showing that RA self-assembles into horizontal dimer monolayers across the graphene surface (Figure 1c, Figure 2b,d). Besides molecular structural configuration, the charge transfer within the graphene/RA hybrid has also been estimated via ab initio calculations. The results indicate that electrons are transferred from graphene to RA molecules, resulting in p-doped graphene. The most stable structure α (Figure 2) leads to a charge density of 1.10 × 10^13^ cm^−2^ (1.17 × 10^13^ cm^−2^) which was estimated via Mulliken population analysis (Hirshfeld method) [19–21].

**Figure 3:**
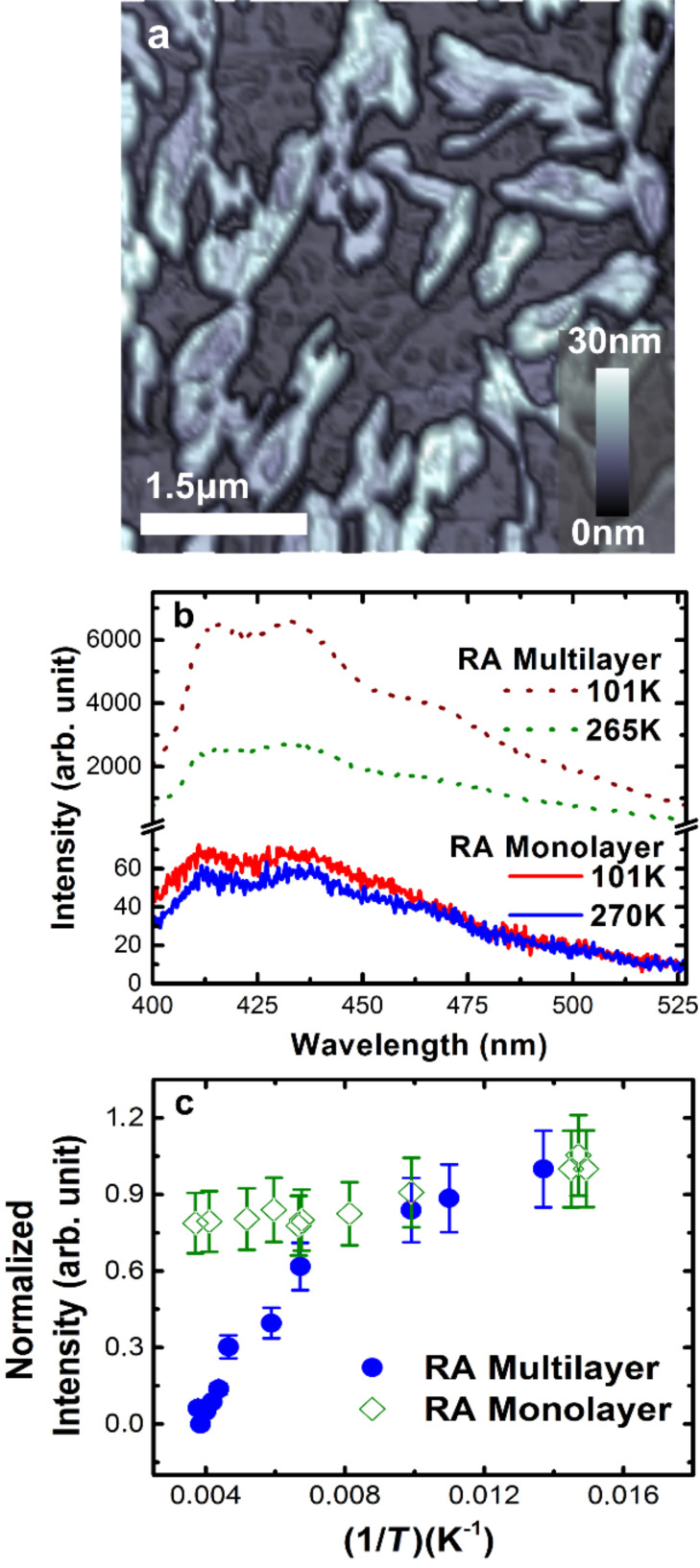
Optical characterization of RA monolayer and multilayers. (a) AFM topographic image of a multilayered RA sample deposited atop graphite microplate. (b) Photoluminescence spectra of RA monolayer (solid lines) and multilayer (dashed lines) at two different temperatures (≈100 K and ≈270 K). (c) Photoluminescence integrated intensity as a function of the inverse of temperature for RA monolayer (green diamonds) and multilayer (blue circles). The laser excitation power (355 nm) was 1 mW.

Retinoic acid is a well-known dye molecule with potential applications in solar cells [22–24] and, thus, its optical properties are key to the successful development of devices. Since molecular packing and ordering influence optical properties of organic semiconductors [7–13], it is important to investigate whether the graphene-induced ordering affected the optical properties of this novel RA SAM structure. Therefore, photoluminescence (PL) studies of RA monolayers and multilayers atop graphite microplates were performed as a function of temperature (Figure 3). Initially, in order to emulate thick and disordered RA films, a graphite microplate substrate was spread coated using a 2 mM RA solution, producing a multilayered RA sample as shown in Figure 3a. The substrate surface is partially covered by a thick and amorphous RA layer (thickness ranges from 2 nm up to ≈10 nm), which reveals no molecular order in high-resolution AFM images (data not shown), in contrast with the well-organized RA SAM in Figure 1. Representative PL spectra for both RA monolayer (solid lines) and multilayer (dashed lines) acquired at two distinct temperatures (≈100 K and ≈270 K) are shown in Figure 3b. Broad bands at ≈410 nm, ≈430 nm and ≈470 nm are visible in all spectra and may be associated, respectively, with resonance Raman bands from all-*trans*-retinal species [25], and with fluorescence recombination from ^1^Bu+ and ^1^Ag-(ππ*) singlet excited manifolds states [26–27].

Nevertheless, the most interesting feature is shown in Figure 3c, which presents the integrated PL intensity as a function of the inverse temperature for RA monolayer (green diamonds) and multilayer (blue circles). The PL intensity of RA monolayer is essentially constant with temperature. In other words, there is no significant increase on emission efficiency even at low temperatures. On the other hand, the multilayered RA sample portrays a substantial increase in PL intensity as the temperature decreases. These features are understood in light of previous studies, which demonstrated that the exciton mobility in organic semiconductor occurs via both dipole–dipole coupling (Förster transfer) and thermally assisted migration [28]. At low temperatures, the volume probed by an exciton is reduced, which reduces the interaction probability with nonradiative traps or defects, reflecting an increase in the emission efficiency of the organic compound [29]. Moreover, the increase in emission efficiency with decreasing temperatures suggests considerable inter-molecular interactions between RA adjacent layers. At low temperatures, torsional rotations of the molecules will be reduced, increasing both the wavefunction overlap among neighboring molecules and the photoluminescence magnitude [30]. This conventional description is exactly the case for the amorphous multilayered RA sample. However, in the RA monolayer case, the phenomenology is different. As previous works have demonstrated, structural organization in π-conjugated polymer films can improve their electrical [3,31–32] and optical [30,32–33] properties. The interface-induced highly ordered SAM structure leads to a reduction in nonradiative quenching pathways, resulting in a temperature-independent high efficiency optical emission state. Even though the exciton thermal energy is smaller at low temperatures and nonradiative processes would be suppressed, the defect density is already small in such well-organized monolayer films and, thus, defect-mediated nonradiative recombination plays a minor role. As a consequence, there should be no substantial increase in recombination efficiency as the temperature decreases, as observed in Figure 3c.

**Figure 4:**
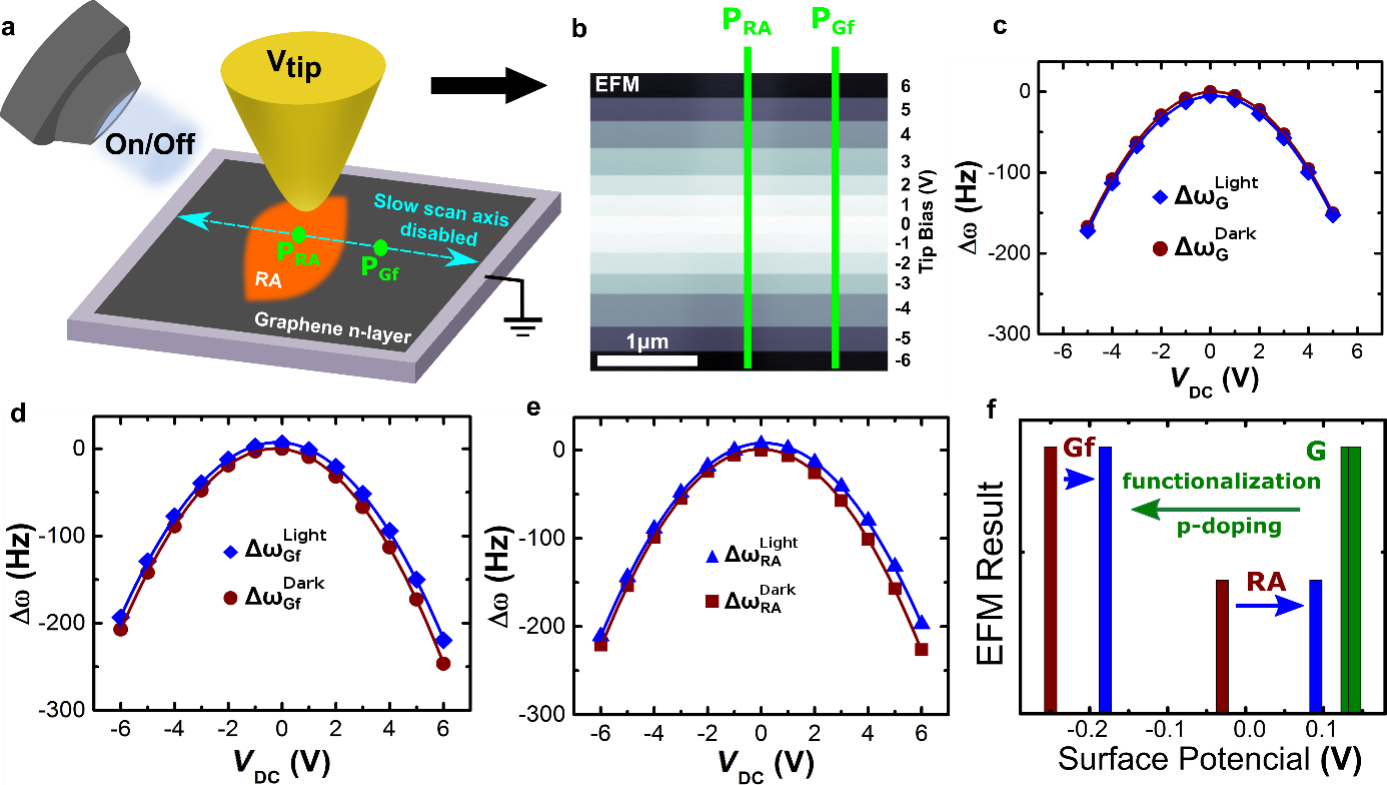
Photo-assisted EFM characterization of the RA/graphene hybrid. (a) Schematic drawing illustrating the photo-assisted EFM experiments. The measurements were performed either in dark (LED off) or under illumination (LED on) on the sample consisting of a graphite microplate substrate (gray) partially covered by RA monolayers (orange). The microscope slow scan axis was disabled and the probe scanned over the same line (blue dashed line) while the image was captured. The bias was applied to the tip (*V*_tip_) and the sample remained grounded during the experiment. The P_RA_ and P_Gf_ points refer to RA monolayer-covered and noncovered regions of the graphite microplate substrate, respectively. All the experiments were performed at ambient conditions. (b) Typical raw EFM image of the photo-assisted experiment. The P_RA_ and P_Gf_ lines refer to RA monolayer-covered and noncovered regions, respectively, where the bias *V*_tip_ varies along the line. The photo-assisted EFM results are summarized in plots of the frequency shift ∆ω at different biases *V*_tip_ for (c) pristine graphene ∆ω_G_ (or graphite microplate – prior to any deposition), (d) functionalized graphene ∆ω_Gf_ (P_Gf_ line) and (e) RA monolayer ∆ω_RA_ (P_RA_ line) in dark (brown curves) and under illumination (blue curves). The brown- and blue-colored symbols in (c), (d) and (e) represent measurements made with and without LED illumination, respectively. The plot in (f) shows the surface potential variation of pristine (G) and functionalized (Gf) graphene/graphite and retinoic acid (RA) SAM upon illumination.

Besides RA SAM optical response, interface-induced modulation of the electrical properties of the RA–graphene hybrid system were investigated in a series of photo-assisted electric force microscopy (EFM) and scanning Kelvin probe microscopy (SKPM) measurements. Figure 4 shows a scheme of the photo-assisted EFM experiments and their results. Initially, a graphite microplate (gray)/RA SAM (orange) sample is electrically connected to the microscope, in which a white LED is mounted and illuminates the sample (Figure 4a). The EFM tip can be biased within the range −6 V < *V*_tip_ < 6 V, creating a strong electric field at the tip apex. In order to analyze the electrical response of the RA/graphene hybrid, the following experimental procedure was employed: during image acquisition, the RA monolayer edge was kept perpendicular to the horizontal (fast scan) direction, while the slow scan (vertical) axis was disabled. In other words, the same region of the RA monolayer and graphite substrate (blue dashed line in Figure 4a) was probed in the entire image. The EFM experiment was carried out at a fixed lift height (50 nm) above the sample surface while the polarization bias *V*_tip_ was varied at finite time intervals, and a typical raw EFM image is shown in Figure 4b. Beginning at the bottom of this image, *V*_tip_ is sequentially increased from −6 V up to +6 V, while the frequency shift ∆ω is recorded (in shades of gray in Figure 4b). The RA monolayer and the functionalized graphene frequency shifts ∆ω_RA_ and ∆ω_Gf_ were extracted, respectively, at the points P_RA_ and P_Gf_ (see green dots in Figure 4a and green lines in Figure 4b; functionalized graphene is defined as a bare surface region of the substrate after RA deposition). Therefore, these green lines refer to extracted data points on RA monolayer and exposed graphene surfaces, respectively, as a function of applied tip bias *V*_tip_. Additionally, for reference purposes, photo-assisted EFM data were also acquired on pristine graphene (∆ω_G_) – a graphite microplate surface prior to any RA deposition – under dark and illuminated conditions (Figure 4c). Figure 4c–e shows plots of EFM frequency shifts ∆ω as a function of applied bias *V*_tip_ for the cases of pristine graphene (Figure 4c – ∆ω_G_^Dark^ and ∆ω_G_^Light^, respectively), functionalized graphene (Figure 4d – ∆ω_Gf_^Dark^ and ∆ω_Gf_^Light^), and the RA monolayer (Figure 4e – ∆ω_RA_^Dark^ and ∆ω_RA_^Light^). Blue (brown) symbols in Figure 4c–e represent data acquired under (no) illumination. A plot enabling a direct comparison of all data for −3 V < *V*_Tip_ < 3 V and their variation according region and illumination condition is shown in Figure S3 in Supporting Information File 1.

In conventional EFM, cantilever oscillation frequency shift (∆ω) can be modeled by

[1]
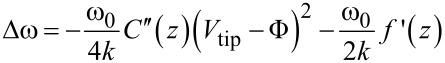


where, ω_0_ and *k* are the cantilever’s resonant frequency and spring constant, respectively, *C*´´(*z*) is the second derivative of the tip–sample capacitance *C*(*z*), *V*_tip_ is the applied bias, Φ is the tip–sample surface potential difference, and *f*´(*z*) is the first derivative of the electric force resulting from permanent polarization or free charges on the surface [34]. In a simpler form, Equation 1 can be rewritten as

[2]
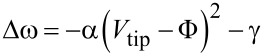


According to Equation 1 and Equation 2, and since all EFM experiments were performed using the same cantilever and at a fixed lift height (fixed capacitance geometry), ∆ω in each region of the image is proportional to the dielectric constant of the material underneath the EFM tip, surface potential differences and to any accumulated charges/permanent polarization at the surface [34]. Equation 2 was used to fit all experimental data in Figure 4c–e (blue or brown solid lines in these plots) and Table 1 shows the respective fitting parameters.

**Table 1:** Fitting parameters of Equation 2 for the experimental data in Figure 4c–e. The error values from the fitting analysis are shown in the first line of the table.

Sample	LED	α ± 0.1 (Hz/V^2^)	Φ ± 0.01 (V)	γ ± 0.2 (Hz)

Graphene (G)	OFF	6.3	0.13	0.2
	ON	6.3	0.14	5.3

Functionalized graphene (Gf)	OFF	6.3	−0.25	−0.3
	ON	5.9	−0.18	−7.4

Retinoic acid (RA)	OFF	6.3	−0.03	−1.1
	ON	5.9	0.09	−7.3

Considering, initially, parameter α in Table 1, which is related to the geometric capacitance and dielectric constant, there is no significant variation, as expected, since the geometric capacitance should remain the same. The minor variation of α for both functionalized graphene (Gf) and RA upon illumination may reflect slight changes on the hybrid dielectric constant upon charge transfer (doping). Such charge transfer, including photo-generated charges, may also account for the observed variation of parameter γ in Table 1, which is related to surface charges [34].

Nevertheless, the most significant information in Table 1 comes from parameter Φ, which is related to tip–sample surface potential differences and is summarized in Figure 4f. This plot schematically shows the surface potential of pristine graphene (graphite microplate surface), G (green color), Gf and RA (brown – dark and blue – illuminated). It is clear in Figure 4f that, upon RA functionalization, the surface potential of a graphite microplate decreases. This provides a signature of p-type doping of graphene, as follows: the work function φ of any material is given by φ = *E*_F_ – *E*_vacuum_, where *E*_F_ is the Fermi energy and *E*_vacuum_ is extracted from the electrostatic potential calculation in the vacuum region near the surface. In the case of electron transfer from graphene to RA molecules (p doping), the graphene Fermi energy moves below the Dirac point, leading to an increase on its work function φ [35–36]. Since the tip–sample surface potential Φ is given by *e*Φ = φ_Tip_ – φ_S_, where φ_Tip_(φ_S_) is the tip (sample) work function and *e* the elementary charge [37], a p-type doping in graphene (sample) increases its work function, hence decreasing the observed surface potential difference Φ of the graphite microplate [36].

**Figure 5:**
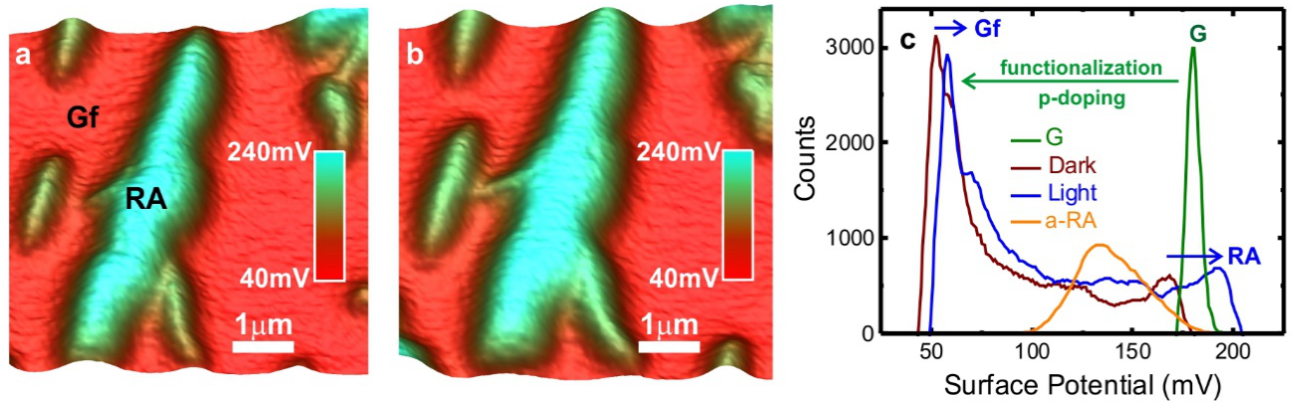
Photo-assisted SKPM characterization of the RA–graphene hybrid. Figures (a) and (b) show SKPM images of the same RA–graphene hybrid region in dark (a) or under illumination (b). In both images, red and green colors indicate functionalized graphene (graphite microplate surface, Gf) and retinoic acid covered (RA) regions, respectively. c) Plot of surface potential histograms of the SKPM images in (a) dark (brown line), (b) light (blue line). Surface potential histograms in dark from a pristine graphite microplate surface (G) – green line – and a thick amorphous retinoic acid layer (a-RA) – orange line – are also shown.

Another important result from Figure 4f is that the surface potential of both Gf and RA increase upon illumination, being more pronounced for the retinoic acid region. Even though the photo-assisted EFM experiments in Figure 4 enable a qualitative analysis of surface potential behavior, their quantitative values may not be as precise, as they might suffer from several approximations leading to Equation 1 and Equation 2 [34]. Therefore, another established SPM-based mode, SKPM, which directly measures surface potential differences [34,37] was employed for a surface potential mapping of the RA–graphene hybrid system and also enabling a direct comparison with the photo-assisted EFM results of Figure 4. Figure 5 summarizes the SKPM analysis (under dark and illuminated conditions) of the RA–graphene hybrid system. Figure 5a,b shows SKPM images of the same region of a hybrid sample in dark and illuminated conditions, respectively (see Figures S4 and S5 for a topographic AFM image of this region and line profiles (topography and surface potential) extracted near the central region of the images, respectively, Supporting Information File 1). In both images, red or green colors indicate functionalized graphene (graphite microplate surface Gf) or retinoic acid covered (RA) regions, respectively. In order to make a statistical analysis of observed values, the surface potential at each image pixel in Figure 5a,b was used to construct a histogram as shown in Figure 5c (brown line – dark; blue line – illuminated). A SKPM image of a pristine graphite microplate was also acquired (Figure S2, Supporting Information File 1) and used to generate its respective histogram, which is also plotted in Figure 5c (green line). Finally, a thick and amorphous RA film (a-RA) covering a graphite substrate was also imaged via SKPM (Figure S2, Supporting Information File 1) and its respective surface potential histogram is shown in Figure 5c (orange line).

Initially, considering the pristine graphite microplate surface case (green line in Figure 5c), the measured histogram is very uniform and there is a surface potential difference Φ ≈ 180 mV between the Au-coated SKPM tip and graphene. This value is in excellent agreement with known values of Au work function (5.10 eV), graphene work function (4.92 eV), and the fact that *e*Φ = φ_Tip_ – φ_S_ [38–39]. When the RA–graphene hybrid is formed, the surface potential difference between Gf (red colors in Figure 5a,b) and the SKPM tip shifts more than 100 mV to lower values either in dark or illuminated conditions. As discussed above for the EFM data, such downshift is a consequence of the increased work function of Gf, which results from the p-doping of Gf induced by RA functionalization. The data in Figure 5 clearly indicate that RA self-assembled monolayers present a larger surface potential difference compared to graphite microplate (in agreement with the photo-assisted EFM experiments in Figure 4). In other words, the work function of RA SAMs is smaller than that of graphene in the hybrid system. It should be noted that, while it is possible to analyze the effect of the RA SAM on the surface potential of graphene or graphite microplate, the opposite is not possible straightforwardly. In the first case, a pristine graphite surface (a graphene without any RA molecules) does exist, enabling a comparison, while in the second case, there is no “pristine RA SAM”. RA SAM is only formed atop graphene or graphite. Nevertheless, a comparison between an organized RA monolayer and a thick amorphous RA film is possible, which could indicate any effect of graphene-induced organization and charge transfer on RA surface potential. Therefore, Figure 5c also portrays the surface potential histogram of a thick amorphous RA film (a-RA) in dark condition. This somewhat broad histogram may reflect several different molecular conformations and surface states within the amorphous RA film (Figure S2, Supporting Information File 1). Nevertheless, comparing its peak position with the peak positions of the RA SAMs (either in dark or illuminated), one observes an upward shift in the surface potential of tens of mV. In other words, upon spatial organization and charge transfer induced by graphene layers, the surface potential of retinoic acid increases. This means that, in the hybrid system, graphene and RA induce opposite surface potential variations on each other, which may be understood simply as signatures of respective RA-induced p-doping of graphene and graphene-induced n-doping of RA resulting from charge transfer.

The effect of illumination on the hybrid system is also portrayed in Figure 4f and Figure 5c. Both photo-assisted EFM and SKPM experiments show an increase on the surface potential of graphene (graphite microplate surface) and RA SAM upon illumination (blue arrows in Figure 4f and Figure 5c). And both techniques also indicate a larger upward shift of the RA SAM surface potential when compared to the graphene potential. The increase of graphene surface potential upon illumination is well-documented in the literature, especially with short wavelengths, like UV light [39–40]. Such increase is associated with photo-electron generation which induces an effective n-doping of graphene, increasing its surface potential [39–40]. The effective n-doping is supposedly achieved when an electron–hole pair is photogenerated and liberates graphene adsorbates via hole recombination (e.g., h^+^ + O_2_^−^ → O_2_ (gas)), releasing electrons that, then, contribute toward an effective n-doping [39–40]. The shorter the wavelength, the more effective such process is [39–40]. Thus, in the present case, which uses white light illumination, the shorter wavelengths should be responsible for most of this effect. The increase on the RA SAM potential might result from concomitant contributions of several mechanisms, including photo-electron generation via hole recombination with RA surface states and adsorbates, photomodification of graphene-RA interfacial dipole, surface band bending and others [39–45]. An analysis of the exact contribution of each mechanism might be very complex and is out of the scope of the present work. The realization of RA SAMs atop other 2D materials and its subsequent photoelectrical characterization may shed some light on the dominant mechanisms.

**Figure 6:**
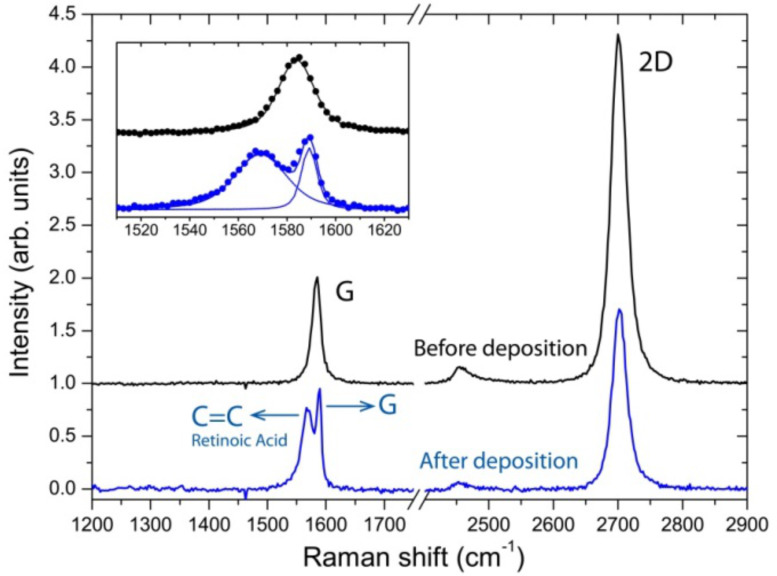
Raman spectroscopy data. Raman spectra obtained from the same region of a graphene monolayer before (black) and after (blue) deposition of RA molecules. For a better visualization, the inset at the top left shows the superposition of the G band obtained for both cases. The Raman mode at ≈1570 cm^−1^ originates from C=C RA molecule bond.

Finally, the charge transfer within the graphene/RA hybrid has also been investigated via Raman scattering experiments. Monolayer graphene, instead of graphite microplate, was chosen for this analysis due to the sensitivity of Raman spectroscopy to chemical doping effects in graphene [46–50]. Therefore, Figure 6 shows Raman spectra obtained from the same region of a monolayer graphene. The upper spectrum (black curve) and the bottom spectrum (blue curve) were obtained before and after the deposition of RA molecules, respectively. Both spectra show two major Raman features: the first-order bond-stretching G band [51] and the two-phonon 2D band [52–53].

The single-Lorentzian shape of the 2D band (≈2690 cm^−1^) reassures that the graphene piece is a single-layer flake [52–53]. The inset in the upper left corner of Figure 6 shows that the graphene G band changes its position from 1584cm^−1^ to 1590 cm^−1^, and its full width at half-maximum of 18 cm^−1^ to 8 cm^−1^, after RA functionalization. That is, the G band frequency increases and its width decreases, indicating graphene doping [46–50,54–57]. Additionally, it is possible to observe the Raman mode at ≈1570 cm^−1^ arising from C=C RA molecule bond [58]. Another strong evidence of chemical doping observed in the Raman spectra shown in Figure 6 is a reduction of about 60% of the ratio between the intensities (peak heights) of the 2D and G bands [46–50,54–56] after the RA deposition. The position of the 2D peak discriminates between electron and hole doping [47,54]: for hole doping, the 2D peak position normally increases, as observed in Figure 6. Based on comparison with literature data [47,54], the graphene charge concentration estimated via Raman scattering experiment is of order of 10^13^ cm^−2^, consistent with the value predicted from ab initio calculations.

## Conclusion

Organic semiconductor dyes and 2D materials are classes of materials which have enormous potential applications by themselves. The possibility of creating hybrid systems, where interfacial effects modulate the final properties, creates even further possibilities in the design and operation of innovative devices. The present work paves a few steps forward in this direction. Here, we have demonstrated a novel configuration of the employed dye: a highly ordered self-assembled monolayer of retinoic acid atop graphene. The RA SAM optical properties indicate that such high molecular ordering reduces the exciton interaction with nonradiative processes, leading to a temperature-independent high emission efficiency. Photo-assisted EFM and SKPM measurements carried out on the hybrid system, along with theoretical calculations and Raman scattering experiments, reveal a well-defined p-doping of graphene n-layer. Moreover, photo-assisted EFM experiments revealed surface potential changes in both hybrid constituents under illumination, which are consistent, among other mechanisms, with photohole–adsorbate recombination at the RA/graphene interface. In summary, the present work may promote new strategies in both organic electronics and 2D material applications due to potential interfacial-induced improvements within their hybrid systems.

## Experimental

High-purity graphite flakes were obtained from Nacional de Grafite Ltd., Itapecerica – Brazil. Retinoic acid (RA) was purchased from Alfa Aesar and was used without further purification. The RA multilayer (bulk-like film) was produced by spread coating method [59–61]. Thin film samples were prepared by spin-coating (2200 rpm) from 0.2 mM and 2 mM tetrahydrofuran solution on a given substrate (monolayer graphene up to graphite microplates prepared via mechanical exfoliation of graphite flakes) [16]. According to recent literature, the expression “graphite microplate” is the accurate term to describe a flake with tens of microns in lateral size and tens, or hundreds, of graphene layers in thickness [62–63], which is exactly the substrate we used for SPM and PL measurements. Monolayer graphene flakes, necessary for achieving the highest sensitivity in Raman experiments [47–48], were deposited on Si oxide or quartz substrates, whereas graphite microplate substrates, used in SPM and PL experiments, were deposited on and electrically connected to Ag-coated metallic substrates. The SPM measurements were performed using a Nanoscope V MultiMode SPM from Bruker and morphological characterization was carried out in either peak force or tapping mode. Besides conventional topographic images, peak force mode yields several concomitant images which map mechanical properties of the sample, like adhesion, elastic modulus, dissipation and others [64]. The adhesion channel monitors tip–sample attractive forces along the imaging process, producing high-resolution adhesion maps [64]. Topographic tapping images were acquired at a setpoint ratio *S* = *A*/*A*_0_ = 0.8–0.9, where *A*_0_ and *A* are free and imaging amplitudes, respectively. For peak force imaging, topographic images were acquired at a peak force *F* = 1 nN. Photo-assisted electrostatic force microscopy (EFM) [65–66] and scanning Kelvin probe microscopy (SKPM) [67] experiments were carried out using a white light emitting diode (LED) source. ScanAsyst – Air (bare Si tip), HQ:NSC18/Cr-Au (Au-coated tip) and HQ:CSC37-CrAu (Au-coated tip) cantilevers from Bruker and MikroMasch were used for morphological (bare tip) and electrical characterization (Au-coated tips), respectively. The ScanAsyst-Air probes have typical resonant frequency ω_0_ = 70 kHz and a spring constant *k* = 0.4 N/m. During operation in peak force mode, they are oscillated at a frequency *f* = 2 kHz with a typical amplitude *A* = 2 nm [64]. The HQ:NSC18/Cr-Au and HQ:CSC37-CrAu probes have typical resonant frequencies ω_0_ = 75 kHz and ω_0_ = 20 kHz; and a spring constant *k* = 2.8 N/m and *k* = 0.3 N/m, respectively. For tapping and EFM imaging, these probes are oscillated near their resonant frequency with amplitude *A* ≈ 20 nm. EFM images were acquired with a lift height *z* = 50 nm within the bias range −6 V < *V*_tip_ < 6 V applied to the probe. The photo-assisted EFM data in Figures 4c–e are plots of averaged frequency shift vs bias from multiple (≈20) measurements. The experimental deviation is very small (≈1 Hz – which is smaller than the symbol size in the graphs). The SKPM imaging was performed in the amplitude mode (AM-SKPM) with an AC bias *V*_AC_ = 2 V applied to the probe at the resonant frequency of the cantilever and a lift height *z* = 20 nm. Steady-state photoluminescence (PL) measurements of RA monolayer/graphene system were done at different temperatures using a liquid He immersion cryostat and temperature controller. The sample excitation was performed using a 355 nm line from a pulsed Nd:YAG laser and the PL detection was made by an Andor spectrometer.

### Ab initio calculations

First-principles calculations were based on density functional theory [68–69] as implemented in the SIESTA code [70–71]. For the exchange-correlation potential, we used the van der Waals density functional (vdW-DF) [72–73] as implemented on the Román-Pérez and Soler scheme [74]. We employed norm-conserving Troullier–Martins [75] pseudopotentials in the Kleinman–Bylander [76] factorized form, and a double-ζ basis set composed of finite-range numerical atomic pseudofunctions enhanced with polarization orbitals. A real-space grid was used with a mesh cutoff of 200 Ry. All geometries were optimized so that the maximum force on any atom is less than 10 meV/Å. We used two supercell types in our calculations. The first one was built with hexagonal symmetry and a lattice parameter of *a* = 23.82 Å. In this case, the molecules are considered isolated atop graphene. We also used a rectangular supercell with sides of 34.95 and 8.64 Å (see Figure 2). In order to compare the energetic stabilities of the RA/graphene configurations, we used the formation energies defined as *E*_f_ = *E*_GM_ − *E*_G_ − *E*_M_, where *E*_GM_ is the total energy of the RA/graphene, *E*_G_ is the total energy of isolated graphene, and *E*_M_ is the total energy of isolated RA molecule. Basis set superposition error (BSSE) has been taken into account in all *E*_f_ values using the counterpoise correction [77].

## Supporting Information

File 1 Supporting Information File 1 contains morphological characterization of retinoic acid deposited on different substrates; morphological and electrical characterization of graphite microplate and retinoic acid self-assembled monolayer and amorphous film; and a zoomed-scale graph of the photo-assisted EFM characterization of the RA/graphene hybrid.
Additional Figures.
