# Supplementary material for: Electro-optical interfacial effects on a graphene/π-conjugated organic semiconductor hybrid system
Source: Beilstein J Nanotechnol. 2018 Mar 23;9:963–74. doi: 10.3762/bjnano.9.90 (PMC5870164; doi:10.3762/bjnano.9.90)
Supplement: File 1 — Supporting Information File 1 contains morphological characterization of retinoic acid deposited on different substrates; morphological and electrical characterization of graphite microplate and retinoic acid self-assembled monolayer and amorphous film; and a zoomed-scale graph of the photo-assisted EFM characterization of the RA/graphene hybrid. Additional Figures. [file Beilstein_J_Nanotechnol-09-963-s001.pdf]

Supporting Information
for
Electro-optical interfacial effects on a graphene/π-conjugated organic semiconductor hybrid system

Karolline A. S. Araujo[1,2], Luiz A. Cury[1], Matheus J. S. Matos[1,3], Thales F. D. Fernandes[1], Luiz G. Cançado[1] and Bernardo R. A. Neves*[1]

Address: [1]Departamento de Física - Universidade Federal de Minas Gerais – UFMG, Belo Horizonte, Brazil and [2]Instituto Federal de Educação, Ciência e Tecnologia de Minas Gerais – IFMG, Ponte Nova, Brazil and [3]Universidade Federal de Ouro Preto – UFOP, Ouro Preto, Brazil

Email: Bernardo R. A. Neves - bernardo@fisica.ufmg.br

* Corresponding author

Additional Figures

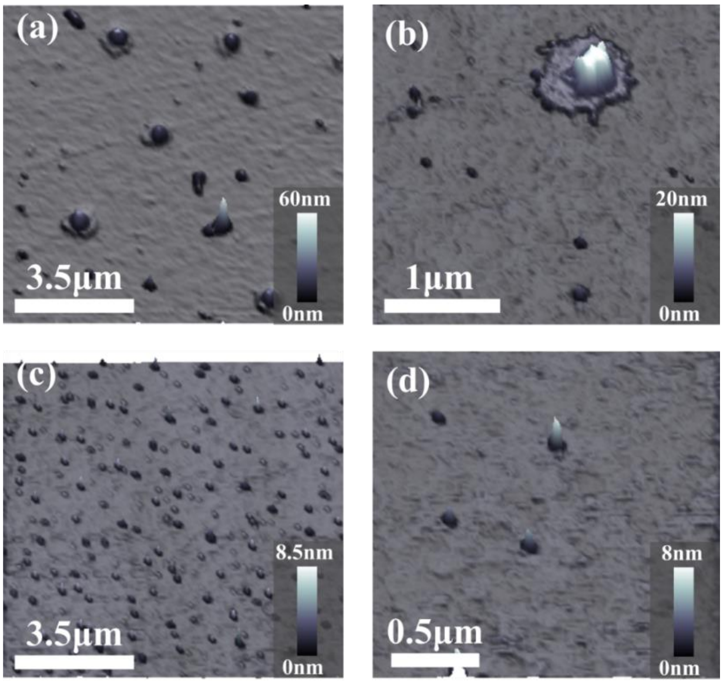

Figure S1: Morphological characterization of retinoic acid (RA) deposited on different substrates. (a)–(b) and (c)–(d) refer to RA spin-coated atop SiO_x and mica, respectively. In all cases, RA forms amorphous agglomerates only. The scale bars and colored scale bars in all images indicate their respective lateral and vertical dimensions.

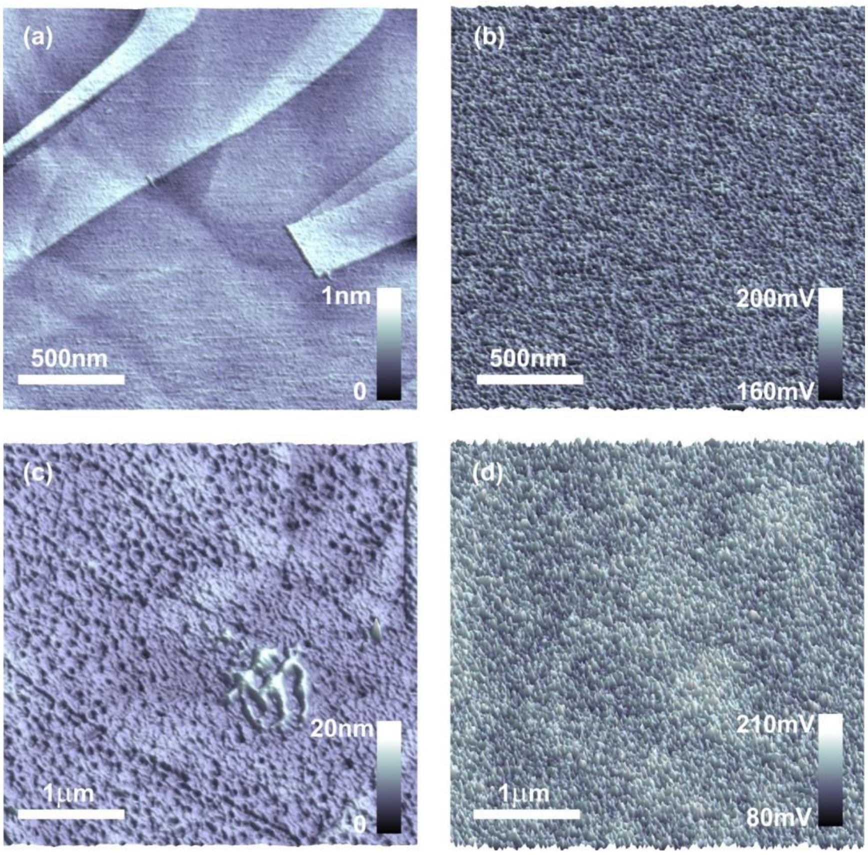

Figure S2: Morphological and electrical characterization of graphite microplate and retinoic acid amorphous film. a) AFM topographic image of a pristine graphite microplate flake. Several different layers (some atomically thin) are seen on this image. b) Scanning Kelvin probe microscopy (SKPM) image of the same region of figure (a). As expected, the pristine flake presents a uniform surface potential across its surface. c) AFM topographic image of a thick and amorphous retinoic acid layer deposited on a graphite microplate. A large amorphous RA agglomerate is seen near the center of the image. Small bubble-like features on the sample surface are due to the solvent evaporation process. d) SKPM image of the same region of figure (c). Slight variations of the surface potential are visible across the image, which present some correlation with topographic features in figure (c). The scale bars and colored scale bars in all images indicate their respective lateral and vertical dimensions.

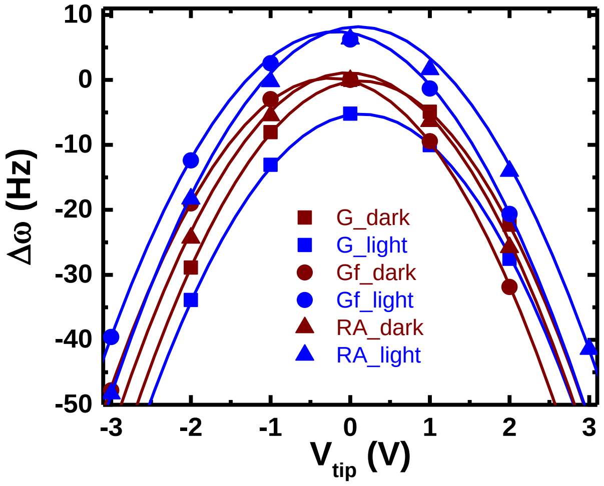

Figure S3: Photo-EFM characterization of the RA/graphene hybrid. Magnified bias scale plot showing photo-EFM results altogether. This plot enables a direct visualization of all data within this bias range and, more specifically, a comparison of the position of each parabola vertex, which is associated with the surface potential, and its variation upon illumination. Brown (blue) symbols indicate experiments carried out in dark (under illumination) conditions. Square symbols refer to pristine graphene (graphite microplate surface) prior to RA deposition, circles refer to bare substrate regions after RA deposition and triangles indicate RA-covered regions. All lines are least-square fits of the experimental data with Equation 2 of the main text.

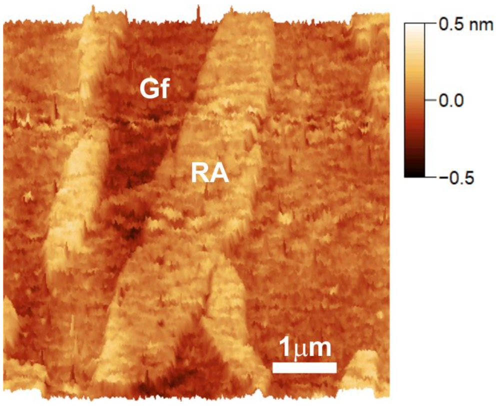

Figure S4: Morphological characterization of the RA-graphene hybrid. RA self-assembled monolayer partially covering a graphite microplate substrate. The SKPM images of Figure 5 in the main text were acquired in this region. The scale bars in the 3D-rendered AFM image indicate its respective lateral and vertical dimensions.

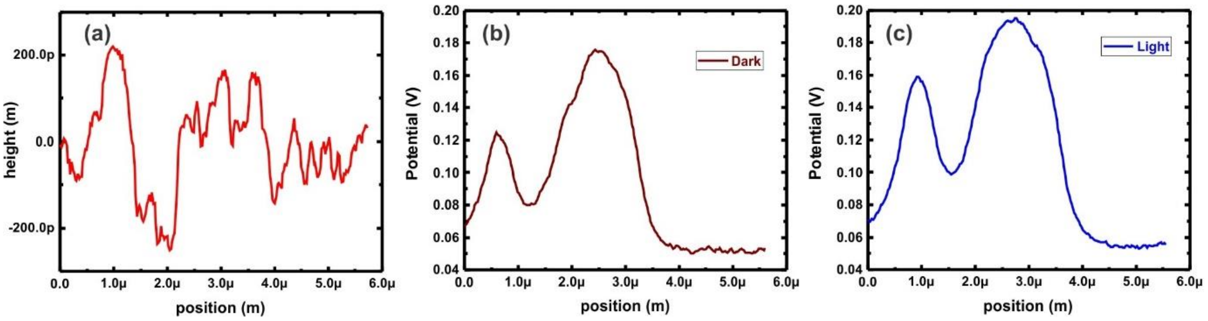

Figure S5: Line profiles (topography (a) and surface potential (b) and (c)) of the same region near the middle of images in Figs. S4 and 5, respectively.
